# The genome sequence of a stonefly, *Nemoura dubitans *(Morton, 1894)

**DOI:** 10.12688/wellcomeopenres.19771.1

**Published:** 2023-08-01

**Authors:** Andrew Farr, Craig R. Macadam, Natural History Museum Genome Acquisition Lab, Darwin Tree of Life Barcoding collective, Wellcome Sanger Institute Tree of Life programme, Wellcome Sanger Institute Scientific Operations: DNA Pipelines collective, Tree of Life Core Informatics collective, Darwin Tree of Life Consortium

**Affiliations:** 1 Independent researcher, Hailsham, England, UK; 2 Buglife – The Invertebrate Conservation Trust, Stirling, England, UK

**Keywords:** Nemoura dubitans, stonefly, genome sequence, chromosomal, Plecoptera

## Abstract

We present a genome assembly from an individual female *Nemoura dubitans* (a stonefly; Arthropoda; Insecta; Plecoptera; Nemouridae). The genome sequence is 321.0 megabases in span. Most of the assembly is scaffolded into 6 chromosomal pseudomolecules, including the X sex chromosome. The mitochondrial genome has also been assembled and is 15.73 kilobases in length.

## Species taxonomy

Eukaryota; Metazoa; Eumetazoa; Bilateria; Protostomia; Ecdysozoa; Panarthropoda; Arthropoda; Mandibulata; Pancrustacea; Hexapoda; Insecta; Dicondylia; Pterygota; Neoptera; Polyneoptera; Plecoptera; Nemouroidea; Nemouridae; Nemourinae; *Nemoura*; *Nemoura dubitans* (Morton, 1894) (NCBI:txid2014036).

## Background

*Nemoura dubitans* is a western Palaearctic species found across central Europe from France to Romania and north to Fennoscandia. It is absent from Wales, Scotland and Ireland, and has a highly localised distribution in the south of England.

It is predominantly found in shallow, heavily vegetated marshes, often where groundwater springs emerge at the surface. The water is often stagnant with large quantities of dead leaves and other rotting material present ( Koese, 2008). There appears to be an association with peat soils with severe records from England being found in fens ( Bratton, 1990). In the western Carpathians *N. dubitans* is found exclusively in fens overgrown with sedges and *Sphagnum* ( Bojková & Helešic, 2009). However, in Slovakia this species is also found in sandy lowland streams and small rivers ( Krno, 2004). In Finland sites with *N. dubitans* were mainly first-order streams with low levels of disturbance by human activities ( Vuori *et al.*, 2006). This species has also been found in reed-lined bays of small lakes in Germany ( Zwick, 2004).

Very little is known about the life history of *Nemoura dubitans*. It is thought that it has a univoltine life cycle with larvae present in the winter and spring ( Graf *et al.*, 2009; Krno, 2004). Adults emerge between April and June in England ( Bratton, 1990; Hynes, 1977).

Larvae of *Nemoura dubitans* feed predominately by gathering and shredding a wide range of autochthonous and allochthonous material ( Graf *et al.*, 2002; Graf *et al.*, 2009). Adults are likely to feed on lichens, fungi, pollen, organic matter and algae ( Hynes, 1977; Tierno de Figueroa & Sánchez-Ortega, 2000).

The genome of the stonefly, *Nemoura dubitans*, was sequenced as part of the Darwin Tree of Life Project, a collaborative effort to sequence all named eukaryotic species in the Atlantic Archipelago of Britain and Ireland. Here we present a chromosomally complete genome sequence for *Namoura dubitans*, based on one female specimen from Surlingham, UK.

## Genome sequence report

**Figure 1.**
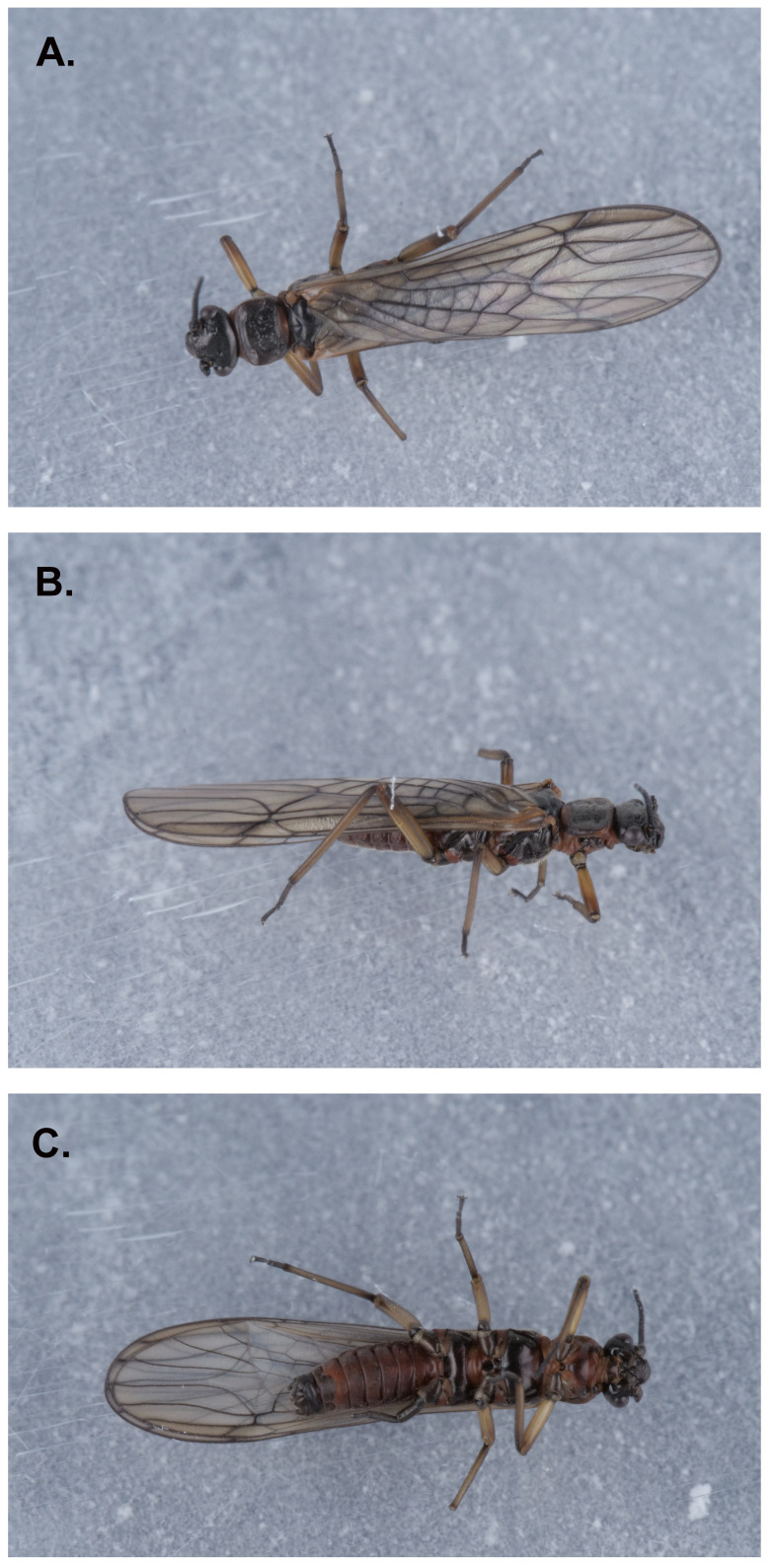
Photograph of the *Nemoura dubitans* (ipNemDubi1) specimen used for genome sequencing.

The genome was sequenced from one female *Nemoura dubitans* ( Figure 1) collected from Surlingham, UK (52.61, 1.41). A total of 62-fold coverage in Pacific Biosciences single-molecule HiFi long reads and 192-fold coverage in 10X Genomics read clouds were generated. Primary assembly contigs were scaffolded with chromosome conformation Hi-C data. Manual assembly curation corrected 54 missing joins or mis-joins and removed one haplotypic duplication, reducing the scaffold number by 28.57%, and increasing the scaffold N50 by 15.84%.

**Figure 2.**
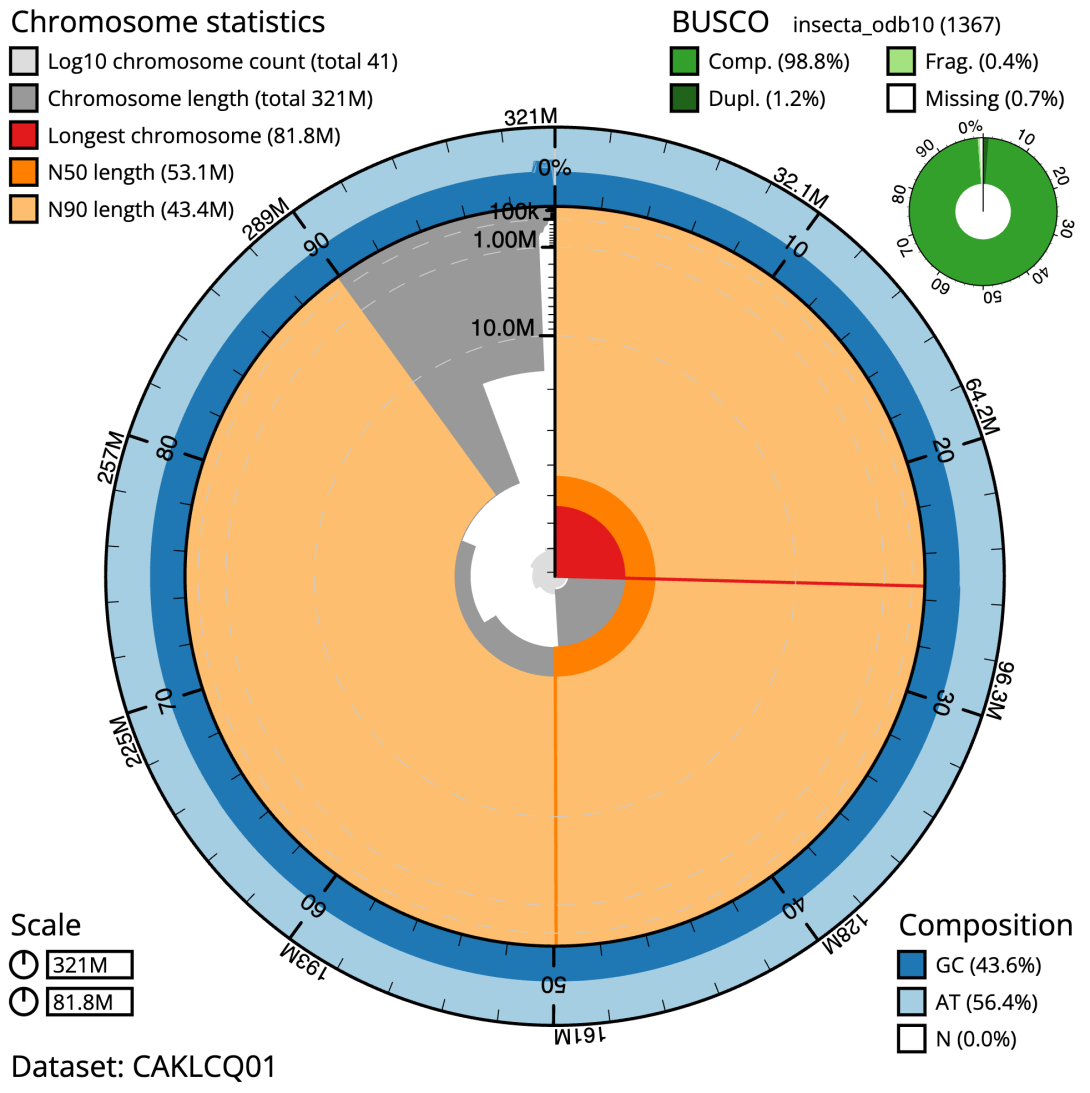
Genome assembly of *Nemoura dubitans*, ipNemDubi1.1: metrics. The BlobToolKit Snailplot shows N50 metrics and BUSCO gene completeness. The main plot is divided into 1,000 size-ordered bins around the circumference with each bin representing 0.1% of the 321,017,140 bp assembly. The distribution of scaffold lengths is shown in dark grey with the plot radius scaled to the longest scaffold present in the assembly (81,771,504 bp, shown in red). Orange and pale-orange arcs show the N50 and N90 scaffold lengths (53,077,913 and 43,408,134 bp), respectively. The pale grey spiral shows the cumulative scaffold count on a log scale with white scale lines showing successive orders of magnitude. The blue and pale-blue area around the outside of the plot shows the distribution of GC, AT and N percentages in the same bins as the inner plot. A summary of complete, fragmented, duplicated and missing BUSCO genes in the insecta_odb10 set is shown in the top right. An interactive version of this figure is available at https://blobtoolkit.genomehubs.org/view/ipNemDubi1.1/dataset/CAKLCQ01/snail.

The final assembly has a total length of 321.0 Mb in 40 sequence scaffolds with a scaffold N50 of 53.1 Mb ( Table 1). Most (99.12%) of the assembly sequence was assigned to 6 chromosomal-level scaffolds, representing 5 autosomes and the X sex chromosome. Chromosome-scale scaffolds confirmed by the Hi-C data are named in order of size ( Figure 2– Figure 5; Table 2). The assignment of chromosome X was based on alignments to the assembly of *Nemurella pictetii* ( Macadam *et al.*, 2022). While not fully phased, the assembly deposited is of one haplotype. Contigs corresponding to the second haplotype have also been deposited. The mitochondrial genome was also assembled and can be found as a contig within the multifasta file of the genome submission.

**Table 1.** Genome data for *Nemoura dubitans*, ipNemDubi1.1.

Project accession data		
Assembly identifier	ipNemDubi1.1	
Species	*Nemoura dubitans*	
Specimen	ipNemDubi1	
NCBI taxonomy ID	2014036	
BioProject	PRJEB46848	
BioSample ID	SAMEA9065873	
Isolate information	ipNemDubi1, female: whole organism (DNA sequencing and Hi-C scaffolding) ipNemDubi2, female: whole organism (RNA sequencing)	
Assembly metrics *		*Benchmark*
Consensus quality (QV)	50.6	*≥ 50*
*k*-mer completeness	99.97%	*≥ 95%*
BUSCO **	C:98.8%[S:97.7%,D:1.2%],F:0.4%,M: 0.7%,n:1,367	*C ≥ 95%*
Percentage of assembly mapped to chromosomes	99.12%	*≥ 95%*
Sex chromosomes	X chromosome	*localised homologous pairs*
Organelles	Mitochondrial genome assembled	*complete single alleles*
Raw data accessions		
PacificBiosciences SEQUEL II	ERR6808018, ERR6808019	
10X Genomics Illumina	ERR6688621–ERR6688624	
Hi-C Illumina	ERR6688620	
PolyA RNA-Seq Illumina	ERR9435016	
Genome assembly		
Assembly accession	GCA_921293005.1	
*Accession of alternate haplotype*	GCA_921293025.1	
Span (Mb)	321.0	
Number of contigs	158	
Contig N50 length (Mb)	5.2	
Number of scaffolds	40	
Scaffold N50 length (Mb)	53.1	
Longest scaffold (Mb)	81.8	

* Assembly metric benchmarks are adapted from column VGP-2020 of “Table 1: Proposed standards and metrics for defining genome assembly quality” from ( Rhie *et al.*, 2021). ** BUSCO scores based on the insecta_odb10 BUSCO set using v5.3.2. C = complete [S = single copy, D = duplicated], F = fragmented, M = missing, n = number of orthologues in comparison. A full set of BUSCO scores is available at https://blobtoolkit.genomehubs.org/view/ipNemDubi1.1/dataset/CAKLCQ01/busco.

**Figure 3.**
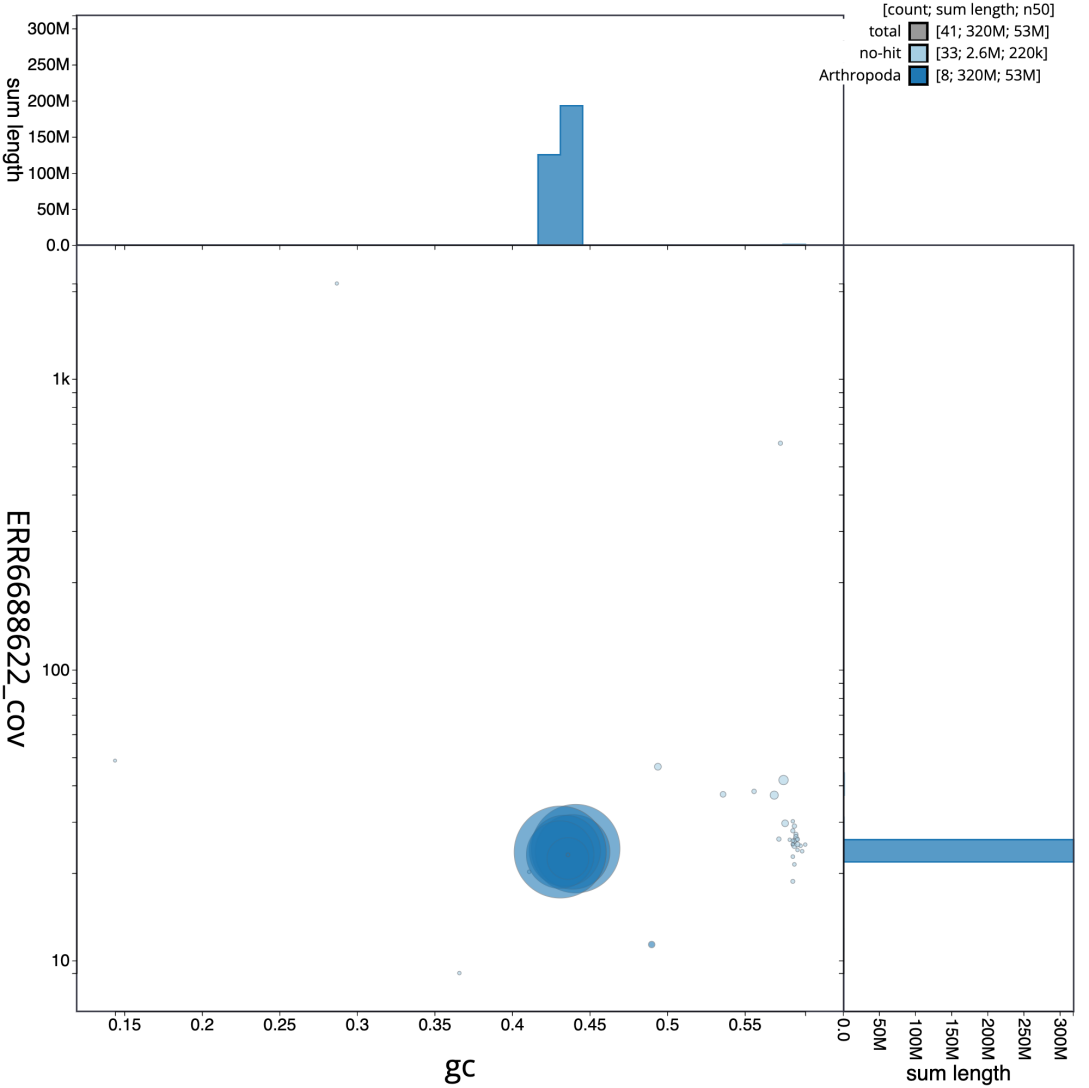
Genome assembly of *Nemoura dubitans*, ipNemDubi1.1: BlobToolKit GC-coverage plot. Scaffolds are coloured by phylum. Circles are sized in proportion to scaffold length. Histograms show the distribution of scaffold length sum along each axis. An interactive version of this figure is available at https://blobtoolkit.genomehubs.org/view/ipNemDubi1.1/dataset/CAKLCQ01/blob.

**Figure 4.**
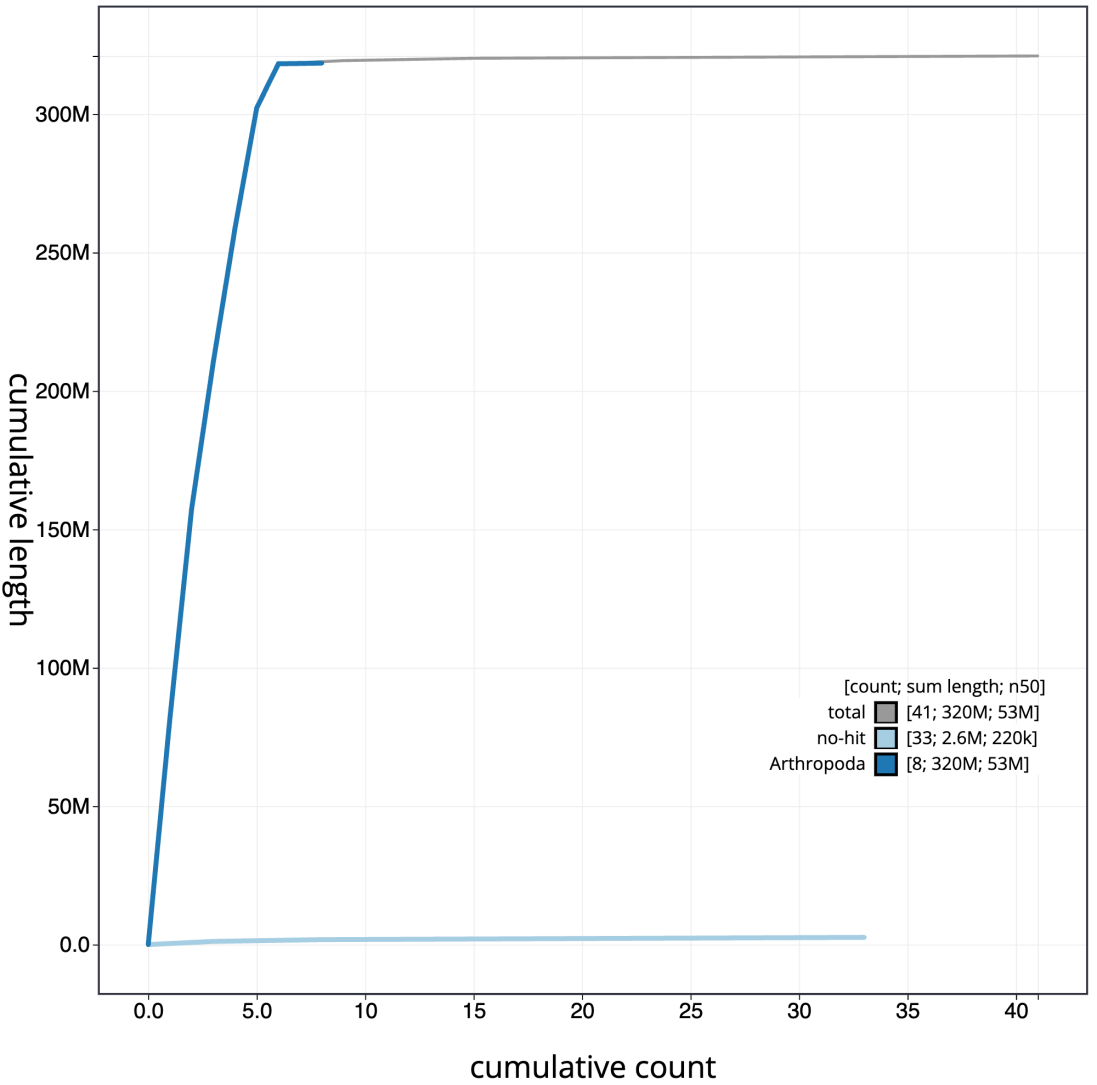
Genome assembly of *Nemoura dubitans*, ipNemDubi1.1: BlobToolKit cumulative sequence plot. The grey line shows cumulative length for all scaffolds. Coloured lines show cumulative lengths of scaffolds assigned to each phylum using the buscogenes taxrule. An interactive version of this figure is available at https://blobtoolkit.genomehubs.org/view/ipNemDubi1.1/dataset/CAKLCQ01/cumulative.

**Figure 5.**
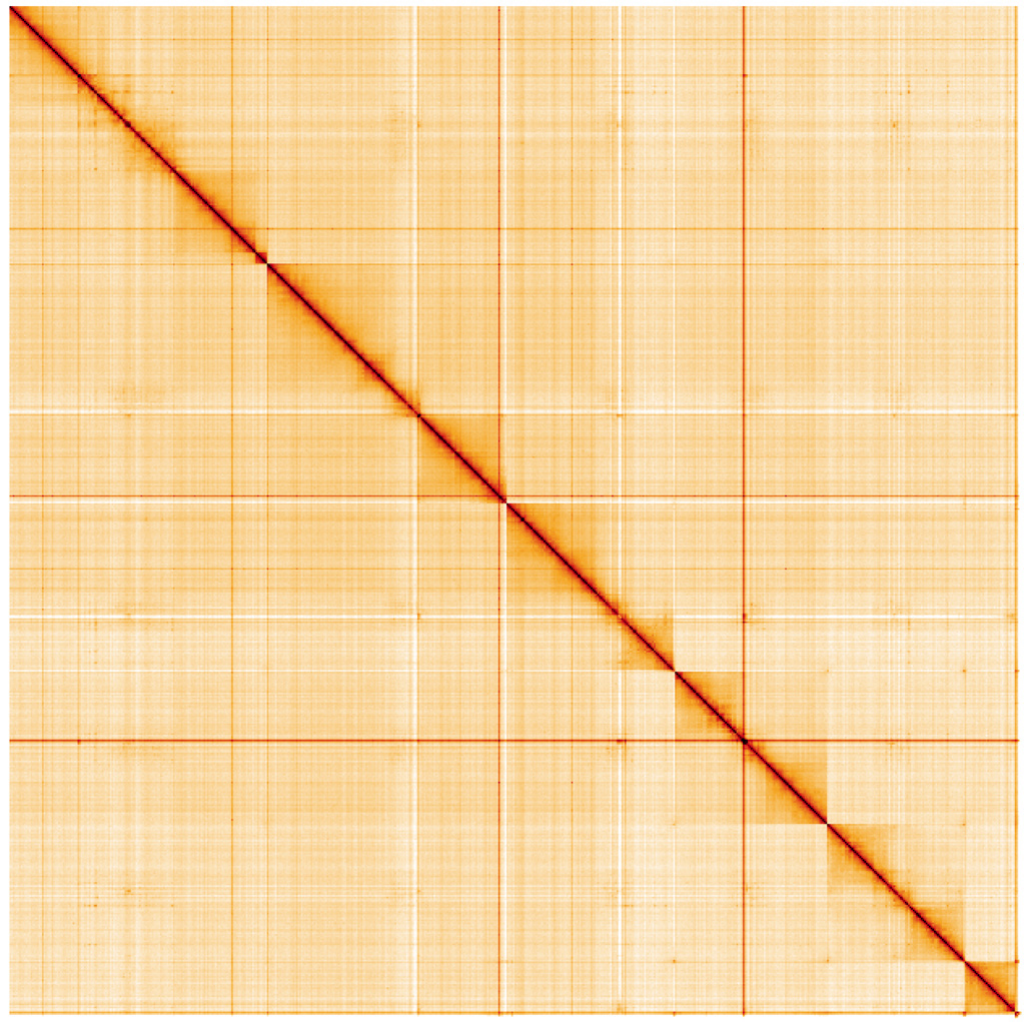
Genome assembly of *Nemoura dubitans*, ipNemDubi1.1: Hi-C contact map of the ipNemDubi1.1 assembly, visualised using HiGlass. Chromosomes are shown in order of size from left to right and top to bottom. An interactive version of this figure may be viewed at https://genome-note-higlass.tol.sanger.ac.uk/l/?d=R0xG0doeQM-knjc_MG2W5A.

**Table 2.** Chromosomal pseudomolecules in the genome assembly of *Nemoura dubitans*, ipNemDubi1.

INSDC accession	Chromosome	Length (Mb)	GC%
OV121074.1	1	75.62	44
OV121075.1	2	53.08	44
OV121076.1	3	48.36	43
OV121077.1	4	43.41	43
OV121078.1	5	15.96	44
OV121073.1	X	81.77	43
OV121079.1	MT	0.02	29

The estimated Quality Value (QV) of the final assembly is 50.6 with *k*-mer completeness of 99.97%, and the assembly has a BUSCO v5.3.2 completeness of 98.8% (single = 97.7%, duplicated = 1.2%), using the insecta_odb10 reference set ( *n* = 1,367).

Metadata for specimens, spectral estimates, sequencing runs, contaminants and pre-curation assembly statistics can be found at https://links.tol.sanger.ac.uk/species/2014036.

## Methods

### Sample acquisition and nucleic acid extraction

The specimen used for genome sequencing was a female *Nemoura dubitans* (specimen ID NHMUK014361789, ToLID ipNemDubi1), collected from a river in Surlingham, UK (latitude 52.61, longitude 1.41) on 2019-04-11. A second female *Nemoura dubitans* was used for RNA sequencing (specimen ID NHMUK014361797, ToLID ipNemDubi2). Both specimens were collected and identified by Andrew Farr (Riverfly Recording Scheme) and dry frozen.

DNA was extracted at the Tree of Life laboratory, Wellcome Sanger Institute (WSI). The ipNemDubi1 sample was weighed and dissected on dry ice with tissue set aside for Hi-C sequencing. Tissue from the whole organism was disrupted using a Nippi Powermasher fitted with a BioMasher pestle *.* High molecular weight (HMW) DNA was extracted using the Qiagen MagAttract HMW DNA extraction kit. Low molecular weight DNA was removed from a 20 ng aliquot of extracted DNA using the 0.8X AMpure XP purification kit prior to 10X Chromium sequencing; a minimum of 50 ng DNA was submitted for 10X sequencing. HMW DNA was sheared into an average fragment size of 12–20 kb in a Megaruptor 3 system with speed setting 30. Sheared DNA was purified by solid-phase reversible immobilisation using AMPure PB beads with a 1.8X ratio of beads to sample to remove the shorter fragments and concentrate the DNA sample. The concentration of the sheared and purified DNA was assessed using a Nanodrop spectrophotometer and Qubit Fluorometer and Qubit dsDNA High Sensitivity Assay kit. Fragment size distribution was evaluated by running the sample on the FemtoPulse system.

RNA was extracted from whole organism tissue of ipNemDubi2 in the Tree of Life Laboratory at the WSI using TRIzol, according to the manufacturer’s instructions. RNA was then eluted in 50 μl RNAse-free water and its concentration assessed using a Nanodrop spectrophotometer and Qubit Fluorometer using the Qubit RNA Broad-Range (BR) Assay kit. Analysis of the integrity of the RNA was done using Agilent RNA 6000 Pico Kit and Eukaryotic Total RNA assay.

### Sequencing

Pacific Biosciences HiFi circular consensus and 10X Genomics read cloud DNA sequencing libraries were constructed according to the manufacturers’ instructions. Poly(A) RNA-Seq libraries were constructed using the NEB Ultra II RNA Library Prep kit. DNA and RNA sequencing were performed by the Scientific Operations core at the WSI on Pacific Biosciences SEQUEL II (HiFi), Illumina HiSeq 4000 (RNA-Seq) and Illumina NovaSeq 6000 (10X) instruments. Hi-C data were also generated from tissue of ipNemDubi1 using the Arima2 kit and sequenced on the Illumina NovaSeq 6000 instrument.

### Genome assembly, curation and evaluation

Assembly was carried out with Hifiasm ( Cheng *et al.*, 2021) and haplotypic duplication was identified and removed with purge_dups ( Guan *et al.*, 2020). One round of polishing was performed by aligning 10X Genomics read data to the assembly with Long Ranger ALIGN, calling variants with FreeBayes ( Garrison & Marth, 2012). The assembly was then scaffolded with Hi-C data ( Rao *et al.*, 2014) using SALSA2 ( Ghurye *et al.*, 2019). The assembly was checked for contamination and corrected using the gEVAL system ( Chow *et al.*, 2016) as described previously ( Howe *et al.*, 2021). Manual curation was performed using gEVAL, HiGlass ( Kerpedjiev *et al.*, 2018) and Pretext ( Harry, 2022). The mitochondrial genome was assembled using MitoHiFi ( Uliano-Silva *et al.*, 2022), which runs MitoFinder ( Allio *et al.*, 2020) or MITOS ( Bernt *et al.*, 2013) and uses these annotations to select the final mitochondrial contig and to ensure the general quality of the sequence.

A Hi-C map for the final assembly was produced using bwa-mem2 ( Vasimuddin *et al.*, 2019) in the Cooler file format ( Abdennur & Mirny, 2020). To assess the assembly metrics, the *k*-mer completeness and QV consensus quality values were calculated in Merqury ( Rhie *et al.*, 2020). This work was done using Nextflow ( Di Tommaso *et al.*, 2017) DSL2 pipelines “sanger-tol/readmapping” ( Surana *et al.*, 2023a) and “sanger-tol/genomenote” ( Surana *et al.*, 2023b). The genome was analysed within the BlobToolKit environment ( Challis *et al.*, 2020) and BUSCO scores ( Manni *et al.*, 2021; Simão *et al.*, 2015) were calculated.

Table 3 contains a list of relevant software tool versions and sources.

**Table 3.** Software tools: versions and sources.

Software tool	Version	Source
BlobToolKit	4.0.7	https://github.com/blobtoolkit/blobtoolkit
BUSCO	5.3.2	https://gitlab.com/ezlab/busco
FreeBayes	1.3.1-17- gaa2ace8	https://github.com/freebayes/freebayes
Hifiasm	0.15.3	https://github.com/chhylp123/hifiasm
HiGlass	1.11.6	https://github.com/higlass/higlass
Long Ranger ALIGN	2.2.2	https://support.10xgenomics.com/genome-exome/ software/pipelines/latest/advanced/other-pipelines
Merqury	MerquryFK	https://github.com/thegenemyers/MERQURY.FK
MitoHiFi	2	https://github.com/marcelauliano/MitoHiFi
PretextView	0.2	https://github.com/wtsi-hpag/PretextView
purge_dups	1.2.3	https://github.com/dfguan/purge_dups
SALSA	2.2	https://github.com/salsa-rs/salsa
sanger-tol/genomenote	v1.0	https://github.com/sanger-tol/genomenote
sanger-tol/readmapping	1.1.0	https://github.com/sanger-tol/readmapping/tree/1.1.0

### Wellcome Sanger Institute – Legal and Governance

The materials that have contributed to this genome note have been supplied by a Darwin Tree of Life Partner. The submission of materials by a Darwin Tree of Life Partner is subject to the **‘Darwin Tree of Life Project Sampling Code of Practice’**, which can be found in full on the Darwin Tree of Life website here. By agreeing with and signing up to the Sampling Code of Practice, the Darwin Tree of Life Partner agrees they will meet the legal and ethical requirements and standards set out within this document in respect of all samples acquired for, and supplied to, the Darwin Tree of Life Project. 

Further, the Wellcome Sanger Institute employs a process whereby due diligence is carried out proportionate to the nature of the materials themselves, and the circumstances under which they have been/are to be collected and provided for use. The purpose of this is to address and mitigate any potential legal and/or ethical implications of receipt and use of the materials as part of the research project, and to ensure that in doing so we align with best practice wherever possible. The overarching areas of consideration are:

•   Ethical review of provenance and sourcing of the material

•   Legality of collection, transfer and use (national and international) 

Each transfer of samples is further undertaken according to a Research Collaboration Agreement or Material Transfer Agreement entered into by the Darwin Tree of Life Partner, Genome Research Limited (operating as the Wellcome Sanger Institute), and in some circumstances other Darwin Tree of Life collaborators.

## Data Availability

European Nucleotide Archive: *Nemoura dubitans*. Accession number PRJEB46848; https://identifiers.org/ena.embl/PRJEB46848. ( Wellcome Sanger Institute, 2021) The genome sequence is released openly for reuse. The *Nemoura dubitans* genome sequencing initiative is part of the Darwin Tree of Life (DToL) project. All raw sequence data and the assembly have been deposited in INSDC databases. The genome will be annotated using available RNA-Seq data and presented through the Ensembl pipeline at the European Bioinformatics Institute. Raw data and assembly accession identifiers are reported in Table 1.

## Author information

Members of the Natural History Museum Genome Acquisition Lab are listed here: https://doi.org/10.5281/zenodo.4790042.

Members of the Darwin Tree of Life Barcoding collective are listed here: https://doi.org/10.5281/zenodo.4893703.

Members of the Wellcome Sanger Institute Tree of Life programme are listed here: https://doi.org/10.5281/zenodo.4783585.

Members of Wellcome Sanger Institute Scientific Operations: DNA Pipelines collective are listed here: https://doi.org/10.5281/zenodo.4790455.

Members of the Tree of Life Core Informatics collective are listed here: https://doi.org/10.5281/zenodo.5013541.

Members of the Darwin Tree of Life Consortium are listed here: https://doi.org/10.5281/zenodo.4783558.
