# Supplementary material for: The genome sequence of a stonefly, Nemoura dubitans (Morton, 1894)
Source: Wellcome Open Res. 2023 Aug 1;8:329. [Version 1] doi: 10.12688/wellcomeopenres.19771.1 (PMC11252649; doi:10.12688/wellcomeopenres.19771.1)
Supplement: Peer review history

# Reviewer report: Reviewer response for version 1

**DOI:** 10.21956/wellcomeopenres.21898.r85885

**Published:** 2024-07-16

**Authors:** Siegfried Roth

**Affiliations:** 1 University of Cologne, Cologne, Germany

As background for presenting the genome assembly the authors describe the ecology and distribution of the stonefly, *Nemoura dubitans*. In addition, the authors could have provided some context on the phylogenetic position of the stoneflies within the Polyneoptera, which has been recently revised ( ^1^). They also could have mentioned the two other stonefly genomes produced by the Darwin Tree of life consortium ( ^2^, ^3^) highlighting similarities and differences.

Are sufficient details of methods and materials provided to allow replication by others?

Yes

Is the rationale for creating the dataset(s) clearly described?

Yes

Are the datasets clearly presented in a useable and accessible format?

Yes

Are the protocols appropriate and is the work technically sound?

Yes

Reviewer Expertise:

Evolutionary developmental biology

I confirm that I have read this submission and believe that I have an appropriate level of expertise to confirm that it is of an acceptable scientific standard.

## References

[1] : Evolutionary history of Polyneoptera and its implications for our understanding of early winged insects. *Proc Natl Acad Sci U S A* .2019;116(8) : 10.1073/pnas.1817794116 3024-3029 10.1073/pnas.1817794116 30642969 PMC6386694

[2] : The genome sequence of a stonefly, Nemurella pictetii Klapalek, 1900. *Wellcome Open Res* .2022;7: 10.12688/wellcomeopenres.17684.2 56 10.12688/wellcomeopenres.17684.2 35592548 PMC9086528

[3] : The genome sequence of the black needle fly, Leuctra nigra (Olivier, 1811). *Wellcome Open Res* .2023;8: 10.12688/wellcomeopenres.19082.1 95 10.12688/wellcomeopenres.19082.1 37346817 PMC10280035

# Reviewer report: Reviewer response for version 1

**DOI:** 10.21956/wellcomeopenres.21898.r85881

**Published:** 2024-06-18

**Authors:** Marta Coronado-Zamora

**Affiliations:** 1 Institute of Evolutionary Biology, Barcelona, Spain

This data note describes a high quality chromosomal assembly of the stonefly *Nemoura dubitans *using appropriate sequencing and assembly methods. The genome data are available at ENA, and data is accessible.

**Small comments**

 - RNA-seq is also generated for this species, however, this is not mentioned in the Results section. 

 - The article could benefit from expanding the background with a couple of sentences on the objectives and applications, besides being sequenced as a part of a consortium.  If I'm not mistaken, this is the first genome available for this species, this could be mentioned too. 

 - Mention the sex determination of this specifies could be useful

 - The second female was also collected in the same location and on the same date?

 - Specify that these numbers after mentioning the collection site refers to longitude and latitude: (52.61, 1.41).

 - *Nemoura dubitans* could be shortened to *N. dubitans* after the first mention in each section.

 - Inconsistency between the tool name in the text and in Table 3: Pretext vs Pretext View, SALSA vs SALSA 2

 - "bwa-mem 2" is missing from Table 3

 Overall, this new genome resource by the Darwin Tree of Life Consortium is excellent.

Are sufficient details of methods and materials provided to allow replication by others?

Yes

Is the rationale for creating the dataset(s) clearly described?

Partly

Are the datasets clearly presented in a useable and accessible format?

Yes

Are the protocols appropriate and is the work technically sound?

Yes

Reviewer Expertise:

bioinformatics, population genetics, epigenetics, transposable elements, adaptation

I confirm that I have read this submission and believe that I have an appropriate level of expertise to confirm that it is of an acceptable scientific standard.
